# 免疫检查点抑制剂在小细胞肺癌治疗中的应用与临床试验进展

**DOI:** 10.3779/j.issn.1009-3419.2021.102.41

**Published:** 2021-11-20

**Authors:** 惠秋 张, 西阳 李, 西川 李, 延军 苏

**Affiliations:** 1 300387 天津，天津师范大学生命科学学院，天津市动植物抗性重点实验室 Tianjin Key Laboratory of Animal and Plant Resistance, College of Life Sciences, Tianjin Normal University, Tianjin 300387, China; 2 300060 天津，天津医科大学肿瘤医院肺部肿瘤科，天津市肿瘤防治重点实验室 Key Laboratory of Cancer Prevention and Therapy, Department of Lung Cancer, Tianjin Medical University Cancer Institute and Hospital, Tianjin 300060, China

**Keywords:** 肺肿瘤, 免疫治疗, 免疫检查点抑制剂, 临床试验进展, 生物标志物, Lung neoplasms, Immunotherapy, Immune checkpoint inhibitors, Clinical trial progress, Biomarkers

## Abstract

小细胞肺癌（small cell lung cancer, SCLC）是一种神经内分泌型肿瘤，病情进展快、恶性程度高、易复发、预后极差。在过去的30余年中，SCLC的临床治疗策略以化疗和放疗为主，但疗效并不显著；当前SCLC的免疫治疗逐渐进入临床，并取得了一定的进展。肿瘤的免疫治疗包括免疫检查点抑制剂、肿瘤疫苗、细胞因子、嵌合抗原受体T细胞免疫疗法（chimeric antigen receptor T-cell immunotherapy, CAR-T）等，目前以免疫检查点抑制剂的应用最为广泛。本文总结了免疫检查点抑制剂的原理和相关药物，汇总了它们在SCLC治疗中的国内外临床试验进展，综述了该疗法使用的生物标志物，并探讨了其未来发展方向。

## Abstract (en)

Small cell lung cancer (SCLC) is a neuroendocrine tumor with fast progression, high malignancy, easy recurrence, and extremely poor prognosis. In the past 30 years, the clinical treatment strategy of SCLC has been mainly chemotherapy and radiotherapy, but the curative effect is not significant; the current immunotherapy of SCLC has gradually entered the clinic and has made certain progress. Tumor immunotherapy includes immune checkpoint inhibitors, tumor vaccines, cytokines, chimeric antigen receptor T-cell immunotherapy (CAR-T) therapy, *etc*. Currently, immune checkpoint inhibitors are the most widely used. This article summarizes the principles of immune checkpoint inhibitors and related drugs, summarizes their domestic and foreign clinical trials progress in SCLC treatment, reviews the biomarkers used in the therapy, and discusses its future development direction.

肺癌是世界上发病率和死亡率最高的恶性肿瘤之一。*CA Cancer J Clin*杂志发布的2020年全球癌症统计数据^[1]^表明2020年全球范围内约有220万新发病例、180万新增死亡病例。在男性恶性肿瘤发病率以及死亡率中肺癌均占第1位，在女性恶性肿瘤发病率中肺癌占第2位、死亡率第1位。肺癌根据组织病理学类型的不同，可以分为腺癌、鳞癌、大细胞癌、类癌等非小细胞肺癌（non-small cell lung cancer, NSCLC）和小细胞肺癌（small cell lung cancer, SCLC）。其中SCLC是一种来源于支气管上皮细胞的强侵袭性神经内分泌肿瘤，发病率占肺癌总发病率的15%-20%，与患者吸烟史密切相关。根据美国退伍军人肺癌协会的二期分期法，SCLC一般分为局限期（limited-stage, LS）和广泛期（extensive-stage, ES），LS-SCLC患者病变仅限于一侧胸腔，且能被纳入一个放射治疗视野内，其中60%-70%患者在确诊时已处于ES期，病变超过一个胸腔。SCLC患者病情进展快，易发生化疗耐药与复发转移，恶性程度极高^[2]^；患者平均生存期约8个月-13个月，5年生存率不到2%，预后极差，这些都限制了肺癌治疗方法的出现和新药的研发。近年来，科研人员对肿瘤发生发展机制的研究越发深入，并发现肿瘤细胞通过免疫检查点抑制了免疫系统的功能，以此实现免疫逃逸^[3]^。免疫检查点抑制剂（immune checkpoint inhibitor, ICI）能够提高肿瘤患者的生存是因为其可以阻止肿瘤细胞发生免疫逃逸。因此，ICI在SCLC治疗中的应用与临床试验进展将是未来治疗SCLC的热点区域，本文对该部分内容进行了综述，希望能对临床工作和研究提供帮助。

## 1 SCLC的常规治疗方法

手术虽然是治疗恶性肿瘤的主要手段，但也只有I期（T_1-2_N_0_）患者中极少数能进行手术治疗，其他则无法进行手术治疗。经手术治疗后早期肺癌易出现并发症和病情复发。依托泊苷联合卡铂或顺铂已成为LS-SCLC的一线治疗方案，ES-SCLC的一线治疗方案是铂类联合依托泊苷或伊立替康。在经初期治疗后效果良好，但接近95%的患者存在复发的可能。一线化疗后失败或化疗后复发的患者首选拓扑替康，但此方案的治疗效果有限。此外又因拓扑替康反应并不持久且有血液学毒性，这将限制其在许多患者中的使用。化疗虽然可在早期维持，但患者改善率较低，仅为2.8%-7.2%^[4]^。SCLC的肿瘤突变负荷较高，已经有研究证实了高肿瘤突变负荷与良好的免疫效果有关。其次SCLC对化疗敏感，可以促进免疫抗原的释放，增加T细胞反应，这大大增加了免疫治疗联合化疗用于SCLC治疗的可行性。肿瘤的免疫治疗包括ICI、肿瘤疫苗、细胞因子、嵌合抗原受体T细胞免疫疗法（chimeric antigen receptor T-cell immunotherapy, CAR-T）等疗法^[5]^，其中ICI是近年来研究的热点^[6]^。目前的临床证据可以证明ICI可使SCLC患者的预后得以改善，对SCLC的治疗提供了新的潜力。

## 2 ICI的工作原理与作用靶点

肿瘤细胞利用免疫检查点抑制淋巴细胞的功能，使人体免疫系统自我调节能力降低，发生免疫逃逸。调节性T细胞（regulatory T cells, Treg）在阻碍肿瘤细胞发生免疫逃逸中发挥重要的作用。ICI通过“双信号”系统调控Treg细胞的数量和活性^[7]^，最后促使T细胞杀伤肿瘤细胞^[8]^。目前，较为常用的ICI有程序性死亡受体1（programmed cell death 1, PD-1）及其配体（programmed cell death ligand 1, PD-L1）抑制剂和细胞毒性T细胞相关抗原（cytotoxic T lymphocyte-associated antigen 4, CTLA-4）抑制剂^[9]^。

### 2.1 PD-1、PD-L1抑制剂

PD-1是免疫球蛋白，其主要配体PD-L1广泛表达于肿瘤细胞表面^[10]^，PD-1/PD-L1通路在免疫调节中具有重要的作用。当PD-1与其配体结合后，PD-1胞质区ITSM（immunoreceptor tyrosine-based switch motif）结构域中的酪氨酸会发生磷酸化，招募SHP2（SH2 domain-containing protein-tyrosine phosphatase-2）磷酸酶类分子至胞膜内侧，对下游信号发挥负调控作用，同时T细胞的活化受到抑制，Treg细胞增殖，使得机体的免疫功能降低，发生免疫逃逸^[11]^。当ICI与PD-1或PD-L1结合后，通路被阻断，负调控信号无法传递，T细胞便会分化成为成熟T细胞，清除肿瘤细胞。

纳武单抗（Nivolumab）是靶向PD-1的人免疫球蛋白G4单克隆抗体，是最早被美国食品药品监督管理局（Food and Drug Administration, FDA）审批的PD-1抗体阻断剂。Nivolumab与PD-1结合，可以阻断肿瘤细胞的免疫逃逸，提高免疫系统的抑癌能力^[12]^。CheckMate 032研究结果证明，在复发性SCLC三线或者更晚期治疗中Nivolumab具有较好的疗效和耐受性^[13]^。帕博利珠单抗（Pembrolizumab）是默沙东公司推出的PD-1单抗药物，其作用机制类似于Nivolumab，于2014年7月25日正式获得国家药品监督管理总局（National Medical Products Administration, NMPA）批准在中国上市。

2019年，中国批准度伐利尤单抗（Durvalumab）用于NSCLC局部晚期患者化放疗后的巩固治疗，这是中国首个获批的PD-L1免疫抑制剂。2020年Durvalumab单抗与依托泊苷、卡铂或顺铂被FDA批准，联合用于ES-SCLC患者的一线治疗。CASPIAN是研究Durvaluma单抗联合化疗药物或Durvaluma单抗联合Tremelimumab与单用化疗对未经治疗的ES-SCLC患者的试验。结果显示Durvalumab与化疗联用后患者的OS有明显的提高。目前美国国家综合癌症网络（National Comprehensive Cancer Network, NCCN）肿瘤学临床实践指南将Durvalumab作为SCLC一线治疗的首选方案^[14]^，但在中国临床肿瘤学会指南中Durvalumab是SCLC一线治疗的III级推荐。

2020年2月批准阿替利珠（Atezolizumab）联合卡铂和依托泊苷用于ES-SCLC一线治疗。IMpower133试验^[15]^发现Atezolizumab联合化疗可显著延长SCLC患者的总生存期（overall survival, OS）。目前国内研发出的PD-1抑制剂有以下四种。君实生物的特日普利单抗于2018年12月份在中国上市，用于转移性黑色素瘤的二线治疗，目前正在SCLC一线治疗中开展III期临床试验。对于二线系统化疗后复发性经典型霍奇金淋巴瘤，NMPA已批准信达生物的信迪力单抗用于其治疗。2019年5月，被批准用于治疗经典型霍奇金淋巴瘤的卡瑞利珠单抗在中国上市。百济神州公司的替雷利珠单抗，目前正在开展针对替雷利珠单抗与化疗联合治疗一线胃癌和一线食管癌的全球三期临床试验以及针对一线NSCLC的中国三期临床试验。

### 2.2 CTLA-4抑制剂

CTLA-4是T细胞上的一种跨膜受体，能够与B7、CD80、CD86等配体结合，抑制T细胞的激活，诱导和维持T细胞免疫耐受^[7]^。CTLA-4抗体与CTLA-4结合后可刺激免疫细胞增殖、活化，使得机体免疫应答的反应性提高。伊匹单抗（Ipilimumab）属于CTLA-4抑制剂，是新型的T细胞增强剂和免疫系统激活剂^[16]^。CheckMate-032试验是研究Nivolumab单药或联合Ipilimumab治疗复发耐药SCLC的I期试验，该研究可显著改善患者的生存。NCCN将Nivolumab单药或联合Ipilimumab治疗作为复发SCLC二线治疗的推荐方案之一。替西利姆单抗（Tremelimumab）也是一种CTLA-4抑制剂。在CASPIAN试验^[17]^中，该研究结果显示Durvalumab单抗联合EP方案相比于EP单独治疗可明显改善ES-SCLC患者的OS，而在此基础上增加Tremelimumab可给患者带来进一步的生存获益。

### 2.3 其他抑制剂

LAG-3在结构上类似于CD4受体，但与II型主要组织相容性复合体（major histocompatibility complex, MHC）结合的亲和力高于CD4受体^[18]^。LAG-3高表达患者其总体生存情况较LAG-3低表达患者好。2015的一项临床试验^[19]^评估了LAG525联合Spartalizumab在晚期实体瘤中的治疗效果。该试验是LAG-3单抗的第一项临床试验。此外，一些双特异性抗LAG-3/PD-L1单抗也相继研发出来。

TIGIT抑制剂已开展了大量的临床试验，但还未批准上市。2020年美国临床肿瘤学会年会（American Society of Clinical Oncology, ASCO）会议上，CITYSCAPE试验显示出了很好的结果。该试验评估了TIGIT单抗联合Atezolizumab在NSCLC患者中的疗效。

## 3 SCLC的免疫治疗临床试验

近年来，免疫治疗的不断发展，使SCLC患者的预后和生存有了显著提高。FDA批准PD-1/PD-L1抑制剂及CTLA-4抑制剂可用于多种肿瘤的临床治疗，但由于免疫单药治疗可引起严重的不良反应甚至是死亡；响应性低等原因导致这种治疗手段的总体有效率还很低。因此ICI与多种治疗方案联合的治疗策略应运而生，这种肿瘤联合治疗策略将会成为肿瘤治疗的未来方向。

### 3.1 一线治疗

Ipilimumab是最早在ES-SCLC一线治疗中尝试的免疫治疗药物^[20]^。CA184-041对Ipilimumab联合化疗治疗ES-SCLC患者的相关问题进行研究，研究结果显示联合治疗后中位OS显著提高。III期临床研究CA184-156^[21]^继续在SCLC领域开展，入组的1, 132例ES-SCLC患者其初治的体能状态（performance status, PS）评分0分-1分且无中枢神经系统转移、无自身免疫性疾病。研究发现Ipilimumab联合依托泊苷和卡铂患者的OS无显著提高。两组的客观缓解率（objective response rate, ORR）均为62%，1年OS率均为40%。IMPower 133^[22]^试验是一项多中心双盲试验，纳入的403例ES-SCLC患者初治且PS评分为0分-1分，这项试验允许无症状脑转移患者的纳入^[23]^。试验对Atezolizumab联合化疗对比单一化疗后ES-SCLC患者的进行生成情况进行研究。研究结果发现，化疗联合Atezolizumab组患者的中位OS以及中位无进展生存期（progression-free survival, PFS）均有一定的提高^[15]^。然而，联合治疗发生免疫相关不良反应的概率也是相对较高的。2020年NMPA批准Atezolizumab联合化疗可用于ES-SCLC患者的一线治疗。CASPIAN^[24]^研究是一项针对ES-SCLC患者随机、开放的试验，报告了Durvalumab联合标准化疗对比单纯化疗的OS结果。与IMPower 133研究相似，仅与单一化疗组相比，Durvalumab联合化疗组中位OS和中位ORR显著改善。在化疗药物的选择上CASPIAN研究不同于IMPower 133，不再只应用卡铂，而是顺铂和卡铂均纳入了研究。CASPIAN和IMPower 133试验数据表明，化疗联合免疫疗法可提高ES-SCLC患者的生存。2020年1月开展的KEYNOTE-604^[25]^是一项评估Pembrolizumab联合化疗一线治疗ES-SCLC的随机双盲、安慰剂对照的III期试验。Pembrolizumab联合化疗组PFS显著改善，差异具有统计学意义。Pembrolizumab联合化疗组OS虽然有提高但没有明显差异。但今年进行的Nivolumab联合化疗一线治疗SCLC的研究结果表明，Nivolumab联合化疗组相比单纯化疗组明显改善了PFS，中位OS也得到了延长。

### 3.2 维持治疗

一项II期试验纳入了45例ES-SCLC患者，并且要求受试者在化疗4个-6个周期后无疾病进展。在化疗结束后8周内开始Pembrolizumab维持治疗，共2年，评估的主要指标是PFS。中位OS为9.6个月，中位PFS为1.4个月。其结果与单一ICI治疗SCLC患者二线或三线的ORR相似。以上的结果说明Pembrolizumab维持治疗后患者的预后无明显改善。CheckMate 451^[26]^研究者评估了Nivolumab联合Ipilimumab或Nivolumab单药用于化疗进展后ES-SCLC患者的维持治疗，并与维持期单独使用安慰剂进行了比较。结果表明，Nivolumab联合Ipilimumab组和Nivolumab单药组并未显著提高患者的OS。虽然免疫维持治疗在ES-SCLC中的结果并不乐观，但研究者在LS-SCLC也开展了同步放化疗后免疫治疗维持治疗的临床研究^[27]^，期待能有阳性结果出现。

### 3.3 二线或以后的治疗

由于SCLC较高的发病率，一线治疗后几乎半数以上的患者出现了复发。CheckMate 032试验初始非随机组评估了Nivolumab单药以及与不同浓度的Ipilimumab联用对复发SCLC患者中位PFS和中位OS的影响。结果显示1 mg/kg Nivolumab联合3 mg/kg Ipilimumab组的中位PFS和中位OS较其他组高。FDA批准了纳武单抗用于SCLC患者的三线或后期治疗。CheckMate 331共纳入569例铂类一线治疗后复发且PS评分0分-1分的ES-SCLC患者。试验对Nivolumab和标准化疗药物的疗效进行了对比，主要终点是OS，但未达到。KEYNOTE-158^[28]^试验包括107例至少接受二线及以上治疗的ES-SCLC患者，探究Pembrolizumab对复发ES-SCLC患者的疗效。中期数据表明ORR为18.7%，中位OS为9.1个月。KEYNOTE-028^[29]^同样分析了Pembrolizumab对ES-SCLC患者的有效性和安全性。该研究共纳入24例经治、不能接受标准治疗且PD-L1表达阳性的ES-SCLC患者。ORR、PFS、OS分别为33.3%、1.9个月和9.7个月。通过上述试验数据，FDA批准Pembrolizumab单药用于SCLC患者三线治疗。

## 4 免疫治疗生物标志物

分子生物标志物提供了可操作的治疗靶点，与其不同的是，免疫疗法的生物标志物反映了肿瘤与宿主免疫反应之间的相互作用。随着技术的不断提高，肺癌治疗已经是精准治疗的时代，现阶段我们主要的目标是挑选有效及可靠的生物标志物，筛选出适合免疫治疗的目标人群，最大地提高SCLC免疫治疗的疗效。

### 4.1 PD-L1的表达

因在诱导局部发生免疫抑制中PD-L1的表达的重要作用，许多研究者正在评估其能否作为预测免疫治疗效果的生物标志物。各种肿瘤类型的初步证据^[30]^表明，PD-L1的表达与较高的ORR与抗PD-1/PD-L1治疗有关。但KEYNOTE-158 II期研究表明仅可在40% SCLC中可检测到PD-L1的表达^[31]^。同样CheckMate 032试验也不能预测PD-L1表达作为生物标志物的价值。PD-L1的表达作为生物标志物还面临着几个障碍。PD-L1的表达与肿瘤的定位、细胞类型、肿瘤或组织学类型以及疾病的发病时期有关。检测PD-L1表达情况也没有确切的标准。未来还需要做大量的研究来证明PD-L1表达作为生物标志物的可行性。

### 4.2 肿瘤突变负荷（tumor mutational burden, TMB）

TMB指肿瘤细胞基因组中所评估基因的外显子编码区每兆碱基中发生置换和插入/缺失突变的总数，反映了肿瘤细胞内基因突变程度。TMB越大，被免疫系统识别的可能性越大。SCLC因其普遍与吸烟有关、体细胞突变负荷大等特点被看作是免疫治疗的一种理想肿瘤类型^[32]^。CheckMate 032^[33]^是首个研究TMB作为预测性标志物价值的试验，并取得了令人满意的结果。高TMB的SCLC患者疗效均显著增高^[34]^。然而，2017年ASCO一项研究对SCLC和大细胞神经内分泌肺癌的TMB的相关问题进行研究，研究结果显示，SCLC TMB的中位数为9 mut/Mb，第90百分位数的TMB为19.6 mut/Mb。由于这方面的研究较少，检测技术繁琐且费用昂贵，阈值的界定也没有明确的定义等等，这些问题限制了TMB作为预测性生物标志物。

### 4.3 血液肿瘤突变负荷（blood TMB, bTMB）

由于肿瘤突变负荷的检测方法需要使用组织样本，但是晚期肿瘤患者的组织样本不容易获得，为解决这一问题，研究者们研究出了一种基于血液的TMB方法即bTMB。B-F1RST^[35]^最早对bTMB能否作为预测标志物进行了研究，通过研究发现，与较低bTMB组患者相比，高bTMB的患者无论是PFS、OS还是ORR均有明显优势。但这种方法也有一定的限制。首先，循环肿瘤DNA（circulating tumor DNA, ctDNA）的检测平台和方法不同造成结果差异。其次，血液采样之后应立即分析，同时还应提高检测技术的灵敏度，未来也需要大量的III期临床研究验证bTMB作为生物标志物的可行性。

### 4.4 分子分型

根据SCLC中无刚毛鳞甲复合体同源物样1（achaete-scute complex like 1, ASCL1）、神经源分化因子1（neurogenic differentiation factor 1, NEUROD1）、转录共激活因子1（Yes-associated protein 1, YAP1）及2级POU结构域转录因子3（POU domain, class 2, transcription factor 3, POU2F3）表达的不同可将SCLC分为SCLC-A、SCLC-N、SCLC-Y及SCLC-P四种亚型^[36]^，不同亚型SCLC的生物学特点和对药物的敏感性存在差异，其中SCLC-Y是潜在免疫获益的亚型。近期，Gay等^[36]^在SCLC-A、SCLC-N、SCLC-P的基础上提出了高表达抗原提呈相关基因、T细胞炎性基因表达谱（gene expression profile, GEP）的亚型——SCLC-I。与SCLC-Y相比，SCLC-I更加准确地定义了潜在的免疫获益亚型的特征^[37]^。这提示了分子分型作为SCLC免疫治疗生物标志物的可能性。

## 5 总结和展望

近年来，肺癌的治疗方案日益先进，其中免疫治疗取得了一定的成果。在SCLC的治疗上，不论是放化疗联合、免疫单药还是免疫联合等都显示出了令人满意的治疗效果，但仍存在诸多的不足。什么是最佳治疗策略、如何找到最佳的预测生物标志物以及克服免疫耐药将是未来需解决的问题。由于SCLC肿瘤异质性高、取材困难，目前对于SCLC生物标志物的研究进展较为缓慢，一些在NSCLC中有效的标志物并不适用于SCLC。未来应利用生物信息学技术、全基因组测序和表观遗传学预测真正有临床价值的生物标志物。目前研究者正在进行SCLC免疫治疗试验，期待能为SCLC找到最佳治疗策略。

## References

[1] Liu JJ, Zhang S, Li S (2017). Reconsideration of Chinese lung cancer immunological clinical research under the new wave of immunotherapy. Zhongguo Fei Ai Za Zhi.

[2] Zhang Y, Zhang Y, Jia G (2019). Current status and progress of treatment of small cell lung cancer. Zhongguo Yi Yao Zhi Nan.

[3] Pardoll DM (2012). The blockade of immune checkpoints in cancer immunotherapy. Nat Rev Cancer.

[4] Cui XX, Song P, Zhang L (2019). New progress in diagnosis and treatment of small cell lung cancer. Zhongguo Fei Ai Za Zhi.

[5] Zhu YM, Wen SL, Wu SX (2021). Research progress in immunotherapy of small cell lung cancer. Shandong Yi Yao.

[6] Kottschade LA (2019). The future of immunotherapy in the treatment of cancer. Semin Oncol Nurs.

[7] Simpson TR, Li F, Montalvo-Ortiz W (2013). Fc-dependent depletion of tumor-infiltrating regulatory T cells co-defines the efficacy of anti-CTLA-4 therapy against melanoma. J Exp Med.

[8] Yang YN, Wang Y (2020). Current status and future of immunotherapy predictors for small cell lung cancer. Zhongguo Fei Ai Za Zhi.

[9] Xu YY, Zhan P, Song Y (2020). Clinical research progress in immunotherapy of small cell lung cancer. Zhongguo Fei Ai Za Zhi.

[10] Zhu Y, Wu SK (2020). Immune characteristics of small cell lung cancer. Zhongguo Fei Ai Za Zhi.

[11] Toor SM, Nair VS, Decock J (2020). Immune checkpoints in the tumor microenvironment. Semin Cancer Biol.

[12] Larkin J, Hodi FS, Wolchok JD (2015). Combined Nivolumab and Ipilimumab or monotherapy in untreated melanoma. N Engl J Med.

[13] Ready N, Farago AF, Braud FD (2019). Third-line nivolumab monotherapy in recurrent SCLC: CheckMate 032. J Thorac Oncol.

[14] Liao LF, Yang QQ, Chen Y (2020). Research progress of immune checkpoint inhibitor combination therapy in small cell lung cancer. Zhong Liu Yu Fang Yu Zhi Liao.

[15] Horn L, Reck M, Mok T (2016). PS01.57: IMpower133: a phase I/III study of 1L Atezolizumab with Carboplatin and Etoposide in patients with extensive-stage SCLC. J Thorac Oncol.

[16] Lipson EJ, Drake CG (2011). Ipilimumab: an anti-CTLA-4 antibody for metastatic melanoma. Clin Cancer Res.

[17] Paz-Ares L, Chen Y, Reinmuth N (2019). PL02.11 overall survival with Durvalumab plus Etokposide-Platinum in first-line extensive-stage SCLC: Results from the CASPIAN study. J Thorac Oncol.

[18] Anderson AC, Joller N, Kuchroo VK (2016). Lag-3, Tim-3, and TIGIT: Co-inhibitory receptors with specialized functions in immune regulation. Immunity.

[19] Marin-Acevedo JA, Dholaria B, Soyano AE (2018). Next generation of immune checkpoint therapy in cancer: new developments and challenges. BioMed Central.

[20] Reck M, Bondarenko I, Luft A (2013). Ipilimumab in combination with paclitaxel and carboplatin as first-line therapy in extensive-disease-small-cell lung cancer: results from a randomized, double-blind, multicenter phase 2 trial. Ann Oncol.

[21] Liu Q, Ren SX (2021). Research progress in immunotherapy of small cell lung cancer. Tongji Da Xue Xue Bao (Yi Xue Ban).

[22] Horn L, Mansfield AS, Szczęsna A (2018). First-line Atezolizumab plus chemotherapy in extensive-stage small-cell lung cancer. N Engl J Med.

[23] Yang X, Xiao L, Bao YX (2019). Research progress in the treatment of immune checkpoint inhibitors for small cell lung cancer. Xinjiang Yi Xue.

[24] Goldman JW, Dvorkin M, Chen Y (2021). Durvalumab, with or without tremelimumab, plus platinum-etoposide versus platinum-etoposide alone in first-line treatment of extensive-stage small-cell lung cancer (CASPIAN): updated results from a randomised, controlled, open-label, phase 3 trial. Lancet Oncol.

[25] Rudin C, Shen L, Pietanza MC (2017). P2.04-007 KEYNOTE-604: Phase 3 randomized, double-blind trial of pembrolizumab/placebo plus Etoposide/Platinum for extensive stage-SCLC. J Thorac Oncol.

[26] Owonikoko TK, Kim HR, Govindan R (2019). Nivolumab (nivo) plus ipilimumab (ipi), nivo, or placebo (pbo) as maintenance therapy in patients (pts) with extensive disease small cell lung cancer (ED-SCLC) after first-line (1L) platinum-based chemotherapy (chemo): Results from the double-blind, randomized phase III CheckMate451 study. Ann Oncol.

[27] Iams WT, Porter J, Horn L (2020). Immunotherapeutic approaches for small-cell lung cancer. Nat Rev Clin Oncol.

[28] Even C, Delord JP, Price KA (2021). Evaluation of pembrolizumab monotherapy in patients with salivary gland carcinoma in the phase 2 KEYNOTE-158 study. Oral Oncol.

[29] Ott PA, Elez E, Hiret S (2017). Pembrolizumab in patients with extensive-stage small-cell lung cancer: results from the phase Ib KEYNOTE-028 study. J Clin Oncol.

[30] Kim YJ, Keam B, Ock CY (2019). A phase II study of pembrolizumab and paclitaxel in patients with relapsed or refractory small-cell lung cancer. Lung Cancer.

[31] Huang BL, Duan Y (2019). Clinical research progress of immune checkpoint inhibitors in the treatment of small cell lung cancer. Zhongguo Fei Ai Za Zhi.

[32] Zhang S, Liu JJ, Cheng Y (2017). Clinical development of immune checkpoint inhibitors in patients with small cell lung cancer. Zhongguo Fei Ai Za Zhi.

[33] Ready NE, Ott PA, Hellmann MD (2020). Nivolumab monotherapy and nivolumab plus ipilimumab in recurrent small cell lung cancer: Results from the CheckMate 032 randomized cohort. J Thorac Oncol.

[34] Hellmann MD, Callahan MK, Awad MM (2018). Tumor mutational burden and efficacy of nivolumab monotherapy and in combination with ipilimumab in small-cell lung cancer. Cancer Cell.

[35] Socinski M, Velcheti V, Mekhail T (2019). Final efficacy results from B-F1RST, a prospective phase II trial evaluating blood-based tumour mutational burden (bTMB) as a predictive biomarker for atezolizumab (atezo) in 1L non-small cell lung cancer (NSCLC). Ann Oncol.

[36] Gay CM, Stewart CA, Park EM (2021). Patterns of transcription factor programs and immune pathway activation define four major subtypes of SCLC with distinct therapeutic vulnerabilities. Cancer Cell.

[37] Xie MQ, Chu XL, Zhou J (2021). Research progress of immunotherapy-related biomarkers for small cell lung cancer. Zhongguo Ai Zheng Za Zhi.

